# S100A12 Is Part of the Antimicrobial Network against *Mycobacterium leprae* in Human Macrophages

**DOI:** 10.1371/journal.ppat.1005705

**Published:** 2016-06-29

**Authors:** Susan Realegeno, Kindra M. Kelly-Scumpia, Angeline Tilly Dang, Jing Lu, Rosane Teles, Philip T. Liu, Mirjam Schenk, Ernest Y. Lee, Nathan W. Schmidt, Gerard C. L. Wong, Euzenir N. Sarno, Thomas H. Rea, Maria T. Ochoa, Matteo Pellegrini, Robert L. Modlin

**Affiliations:** 1 Department of Microbiology, Immunology and Molecular Genetics, University of California, Los Angeles, Los Angeles, California, United States of America; 2 Division of Dermatology, David Geffen School of Medicine at University of California, Los Angeles, Los Angeles, California, United States of America; 3 Department of Molecular, Cell, and Developmental Biology, University of California, Los Angeles, Los Angeles, California, United States of America; 4 UCLA/Orthopedic Hospital Department of Orthopedic Surgery, David Geffen School of Medicine at University of California, Los Angeles, Los Angeles, California, United States of America; 5 Department of Bioengineering, University of California, Los Angeles, Los Angeles, California, United States of America; 6 Leprosy Laboratory, Oswaldo Cruz Foundation, Rio de Janeiro, Brazil; 7 Department of Dermatology, University of Southern California School of Medicine, Los Angeles, California, United States of America; New Jersey Medical School, UNITED STATES

## Abstract

Triggering antimicrobial mechanisms in macrophages infected with intracellular pathogens, such as mycobacteria, is critical to host defense against the infection. To uncover the unique and shared antimicrobial networks induced by the innate and adaptive immune systems, gene expression profiles generated by RNA sequencing (RNAseq) from human monocyte-derived macrophages (MDMs) activated with TLR2/1 ligand (TLR2/1L) or IFN-γ were analyzed. Weighed gene correlation network analysis identified modules of genes strongly correlated with TLR2/1L or IFN-γ that were linked by the “defense response” gene ontology term. The common TLR2/1L and IFN-γ inducible human macrophage host defense network contained 16 antimicrobial response genes, including S100A12, which was one of the most highly induced genes by TLR2/1L. There is limited information on the role of S100A12 in infectious disease, leading us to test the hypothesis that S100A12 contributes to host defense against mycobacterial infection in humans. We show that S100A12 is sufficient to directly kill *Mycobacterium tuberculosis* and *Mycobacterium leprae*. We also demonstrate that S100A12 is required for TLR2/1L and IFN-γ induced antimicrobial activity against *M*. *leprae* in infected macrophages. At the site of disease in leprosy, we found that S100A12 was more strongly expressed in skin lesions from tuberculoid leprosy (T-lep), the self-limiting form of the disease, compared to lepromatous leprosy (L-lep), the progressive form of the disease. These data suggest that S100A12 is part of an innate and adaptive inducible antimicrobial network that contributes to host defense against mycobacteria in infected macrophages.

## Author Summary

Macrophage antimicrobial activity induced by innate and adaptive immune stimuli is crucial for controlling infection against intracellular pathogens. In order to characterize host defense pathways, we activated human macrophages with innate and adaptive immune stimuli known to induce antimicrobial activity against mycobacteria, identifying a set of 16 antimicrobial response genes. One of these, S100A12, is present in humans, but not mice, has limited studies in infectious disease. By studying leprosy as a model, we found that expression of S100A12 was greater in skin lesions from patients with the self-limiting versus the progressive form of the disease. Furthermore, we show that S100A12 is sufficient to kill mycobacteria and is required for decreasing the relative viability of *M*. *leprae* in infected macrophages.

## Introduction

Intracellular pathogens reside in macrophages, exploiting host metabolic processes, while at the same time inhibiting host defense mechanisms. However, activation of these infected macrophages via the innate and adaptive immune system triggers antimicrobial responses that overcome microbial escape strategies. A major pathway for macrophage activation via the innate immune system is through the recognition of bacterial lipoproteins by Toll-like receptor 2/1 heterodimers (TLR2/1) [1], which triggers an antimicrobial response [2]. The adaptive immune response can also induce antimicrobial activity in macrophages, for example via activation by IFN-γ [3–5]. TLR2/1L and IFN-γ trigger distinct signaling pathways in human macrophages, but converge on a common host defense pathway, involving the vitamin D-dependent induction of antimicrobial peptides [5–7]. A variety of effector mechanisms are induced in activated macrophages to combat mycobacterial infection as part of the cell-autonomous defense [8], yet the matrix of antimicrobial mediators has not been fully explored. The identification of the host defense network by which the innate and adaptive immune systems induce antimicrobial activity in human macrophages is important for understanding mechanisms of resistance versus susceptibility to intracellular bacteria.

Leprosy, a disease caused by the intracellular bacterium *M*. *leprae*, provides an outstanding model to study innate and adaptive host defense mechanisms in humans because the clinical manifestations correlate with the immune response. At one end of a spectrum, tuberculoid leprosy (T-lep) is characterized by restricted bacterial growth, few skin lesions, and low bacilli numbers. In contrast, at the other end of the spectrum, lepromatous leprosy (L-lep) is characterized by systemically disseminated infection, several skin lesions, and high bacilli numbers. Reversal reactions (RR) on the other hand represent a shift from the lepromatous parts of the spectrum, usually in patients with multibacillary borderline lepromatous leprosy, towards the tuberculoid part of the spectrum and are associated with reduction of bacilli numbers in skin lesions. Here, we hypothesized that activation of macrophages via innate and adaptive immune signals induce antimicrobial activity via common and distinct pathways. Thus, we investigated the functional gene programs in TLR2/1L and IFN-γ activated macrophages, representing two major triggers of macrophage antimicrobial pathways, to identify mechanisms of host defense response against intracellular mycobacteria in humans.

## Results

### Transcriptome analysis of the TLR2/1L and IFN-γ inducible gene network

**Fig 1:**
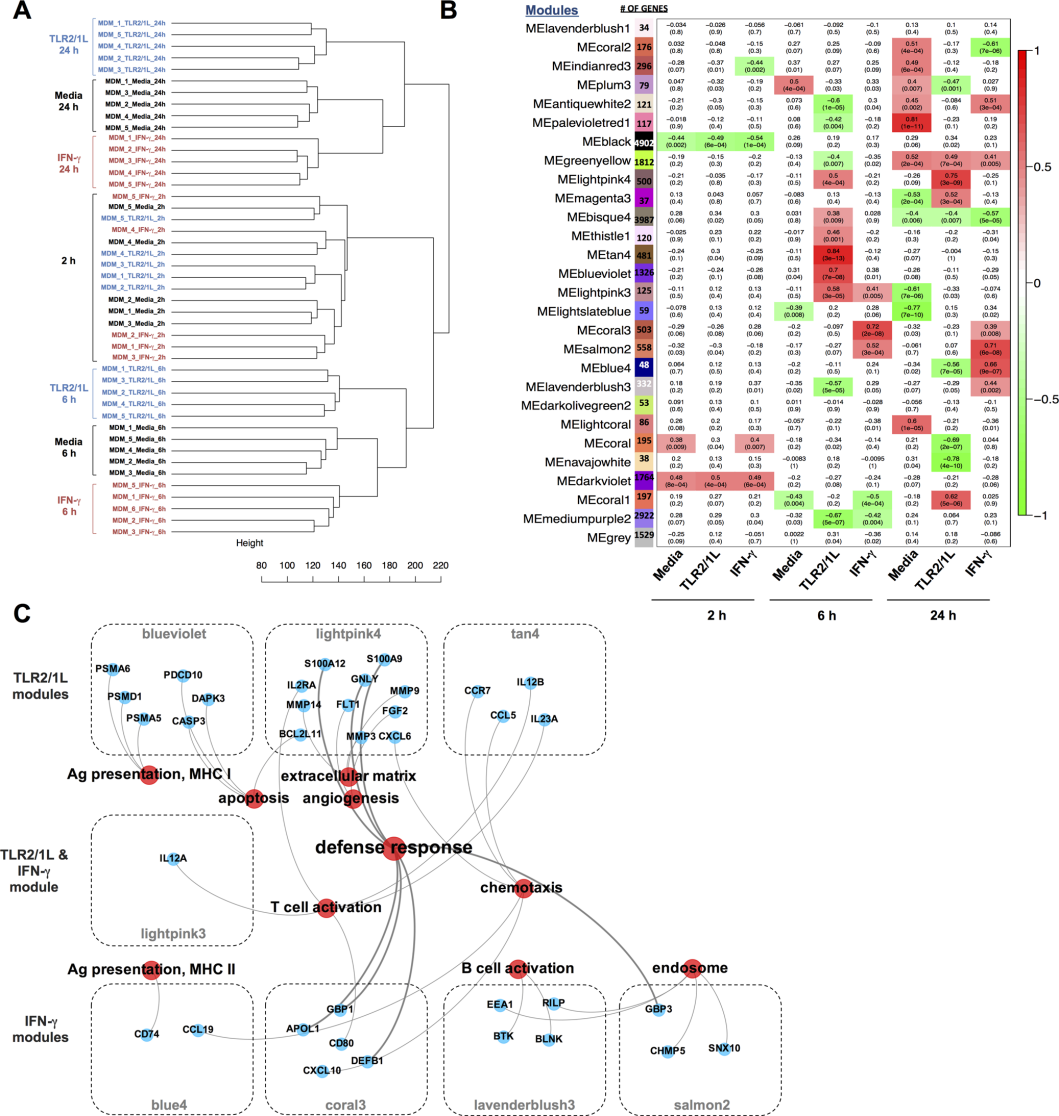
Network analysis of RNAseq reveals defense response genes in TLR2/1 and IFN-γ specific modules. **(A)** Hierarchical clustering of log-transformed normalized counts of 45 samples obtained from RNAseq. For each sample, the replicate number, stimuli, and time point are indicated. **(B)** Correlation of treatment conditions (x-axis) to WGCNA module eigengenes (y-axis) displayed in a heat map. The p-values (bottom) for each r correlation value (top) are indicated for each modules and each condition. Red indicates positive correlation and green indicated an inverse correlation. **(C)** Functional annotation network of significant gene ontology terms (red) from ClueGO analysis associated with representative genes (blue) found in WGCNA modules indicated.

To identify gene networks inducible by both innate and adaptive stimuli, human MDMs from five healthy donors were stimulated with a TLR2/1L (19-kD triacylated lipopeptide derived from mycobacteria), or recombinant human IFN-γ. RNA was collected at 2, 6, and 24 hours and RNAseq was performed. Hierarchical clustering revealed that the media, TLR2/1L and IFN-γ gene expression profiles each clustered separately for the 6 and 24 hour data but were clustered together at 2 hours (Fig 1A). In addition, replicate samples also cluster together, demonstrating the reproducibility of our expression data.

In order to identify genes and pathways associated with the different stimuli, we performed Weight Gene Network Correlation Analysis (WGCNA) on the gene expression profile data [9, 10]. WGCNA is an unsupervised method that uses expression correlations to group genes into modules, similar to traditional clustering analysis, but raises each correlation to a power, thus lending more weight to stronger, more reliable correlations. All samples were analyzed together. WGCNA analysis identified 28 modules of co-expressed genes (Fig 1B, y-axis). To determine which modules were associated with each stimulus and timepoint, we performed module eigengene correlation to the experimental conditions and generated a representative heatmap of correlation values along with their significance (Fig 1B). Since our aim was to identify TLR2/1L and IFN-γ host defense networks, we focused on modules with a positive correlation to these stimuli. At 6 hours, there were several modules that positively correlated with TLR2/1L (‘bisque4’, ‘tristle1’, ‘tan4’, ‘blueviolet’) and one that correlated with both TLR2/1L and IFN-γ (‘lightpink4’). One module correlated with TLR2/1L at both 6 and 24 hours (‘lightpink4’), whereas two correlated with IFN-γ at both time points (‘coral3’, ‘salmon2’). At 24 hours, we identified two TLR2/1L specific modules (‘magenta3’, ‘coral1’) and two IFN-γ specific modules (‘blue4’, ‘lavenderblush3’). At 2 hours, one module (‘black’) positively correlated with all conditions and likely represents a general response to the culture conditions. There were no modules at 2 hours that correlated only with TLR2/1L or IFN-γ and not media, consistent with the finding that all the 2 hour gene expression profile data grouped together by hierarchical clustering.

A total of twelve modules were found to be significantly correlated with TLR2/1L, IFN-γ, or both and not with media control. Functional annotation analysis of each TLR2/1L and IFN-γ linked module was performed using Cytoscape in combination with ClueGO plug-in (S1 Table), which led us to focus on eight modules with gene ontology (GO) terms relevant to the immune response to mycobacteria. Three modules of interest, ‘blueviolet’ and ‘lightpink4’ and ‘tan4’, significantly correlated to TLR2/1L stimulation (correlation = 0.70, 0.75, and 0.84; *P* = 7 x 10^−08^, 3 x 10^−09^, and 3 x 10^−13^ respectively). Four modules, ‘blue4’, ‘coral3’, ‘lavenderblush3’ and ‘salmon2’, were significantly correlated with IFN-γ stimulation (correlation = 0.66, 0.72, 0.44, and 0.71; *P* = 9 x 10^−07^, 2 x 10^−08^, and 6 x 10^−08^ respectively). One module, ‘lightpink3’, was linked to both TLR2/1L and IFN-γ treatment of MDMs (correlation = 0.58 and 0.41; *P* = 2 x 10^−05^ and 5 x 10^−04^, respectively). Two modules yielded no significant GO terms (‘magenta3’ and ‘tristle1’) in our ClueGO analysis. For two other modules (‘coral1’ and bisque4’), we did not identify significantly associated GO terms related to immunological responses, but did find associations with “ephrin receptor signaling” and “translational termination & initiation.” Therefore, these modules were not further studied.

Functional annotation analysis revealed that TLR2/1L and IFN-γ linked modules contained genes enriched with the “defense response” GO term (Fig 1C) totaling 129 genes, 13 of which were antimicrobial-related genes. “Defense response” was associated with antimicrobial genes in the ‘lightpink4’, ‘coral3’, and ‘salmon2’ modules. Two major terms “T cell regulation” and “chemotaxis” were associated with both TLR2/1L and IFN-γ specific modules. “Antigen presentation, MHC I”, “extracellular matrix”, “apoptosis” and “angiogenesis” were preferentially associated with TLR2/1L-induced modules. “Antigen presentation, MHC II”, “B cell activation” and “endosome” were preferentially associated with IFN-γ stimulation. These data indicate that TLR2/1L and IFN-γ induce distinct functional pathways in macrophages, but also common pathways, including a “defense response” network.

### The common TLR2/1 and IFN-γ defense response network

**Fig 2:**
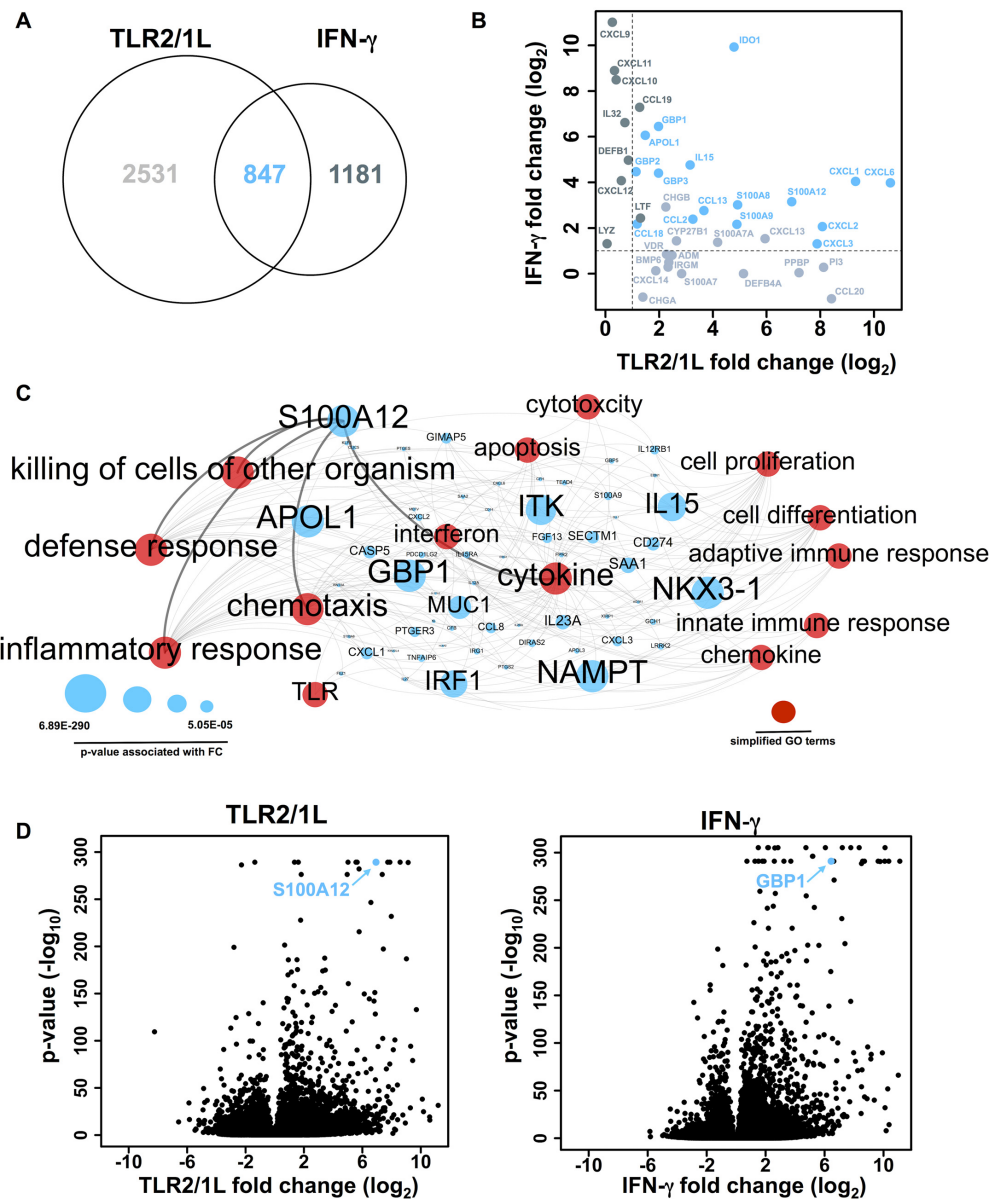
Analysis of TLR2/1L and IFN-γ inducible genes identifies common pathways. **(A)** TLR2/1L and IFN-γ inducible genes defined as fold-change (FC) greater than 2 and *P*-value less than 0.01. Overlap indicates number of genes shared by both stimuli. **(B)** TLR2/1L versus IFN-γ log_2_(FC) of max value for antimicrobial response genes. Genes highlighted in light gray represent TLR2/1L only inducible genes, genes in dark gray represent IFN-γ only inducible genes, and genes in blue represent shared TLR2/1L and IFN-γ inducible genes. **(C)** Functional annotation network of robust TLR2/1L and IFN-γ inducible genes (FC>25 and *P*-value<0.01). Simplified GO terms are in red, genes are in blue. Size of gene node represents *P*-value associated with max FC; largest node has smallest *P*-value in dataset. **(D)** Distribution of max or min log_2_(FC) values versus associated–log_10_(*P*-value) of all genes in dataset. Genes of interest are indicated in blue.

To further define the TLR2/1 and IFN-γ common pathways, gene expression profiles were analyzed using DESeq at each time point to test for differential expression between stimulated versus unstimulated conditions. Differentially regulated genes were defined as having a fold change >2 and adjusted p-value <0.01 in at least one time point. A total of 3,378 TLR2/1L induced genes and 2,024 IFN-γ induced genes were identified, with 847 genes found to be in common (Fig 2A). As expected, TLR2/1L and IFN-γ induced distinct gene profiles. However, there was almost a 20% overlap between TLR2/1L and IFN-γ inducible genes, indicating that these stimuli converge on a common pathway.

The TLR2/1L and IFN-γ inducible genes were overlapped with a curated list of 135 genes with known direct and indirect antimicrobial activity as reported in the literature. The genes which were common to TLR2/1L and IFN-γ treatment conditions included 16 antimicrobial related genes (Fig 2B) including several chemokines and cytokines [11, 12], calgranulins [13, 14], as well as guanylate binding proteins [15, 16]. TLR2/1L and IFN-γ inducible genes that were reported to have direct antimicrobial activity show effects against a broad-spectrum of pathogens, including gram-positive bacteria, gram-negative bacteria, and parasites, but not mycobacteria (S1 Fig). For instance, chemokine CXCL6 has membrane disrupting properties against Gram-postive bacteria including *Streptococcus* spp, Gram-negative bacteria including *Pseudomonas aeruginosa* [17], and parasites such as *Leshmania mexicana* [12]. The calgranulins S100A8, S100A9, and S100A12 also display a broad-spectrum antimicrobial activity in cell-free culture. In addition, there were several antimicrobial genes that were significantly induced by TLR2/1L and not by IFN-γ and vice versa. For example, TLR2/1L induced genes of the S100 protein family, such as S100A7 and S100A7A [18, 19] whereas IFN-γ induced IL32, which encodes a cytokine involved in the IFN-γ vitamin D-dependent antimicrobial response against *M*. *tuberculosis* [10]. These data demonstrate that there are common and distinct antimicrobial pathways in TLR2/1L and IFN-γ activated human macrophages.

To further characterize the common TLR2/1L and IFN-γ induced gene set, we performed gene annotation analysis with ClueGO, which identified significant GO terms associated with “inflammatory response”, “defense response”, “cytokine production”, “cell activation” and “cell chemotaxis” (S2 Table). Within these GO terms, the genes that were robustly induced by either stimulus (FC> 25) were selected and represented in a functional annotation network (Fig 2C). The most significantly induced genes related to “defense response” were S100A12 and GBP1. Within the entire set of gene expression data, S100A12 and GBP1 were among the most strongly and significantly induced genes for TLR2/1L and IFN-γ, respectively (Fig 2D). GBP1 is a member of the guanylate-binding protein family and has been reported to be part of an interferon inducible antimicrobial pathway in macrophages against intracellular bacteria [16, 20], including *Mycobacterium bovis* BCG [15].

S100A12 is a member of the S100 protein family, a group of small cationic proteins characterized by an EF-hand calcium binding motif that are involved in a variety of intracellular and extracellular functions. S100A12 has been reported to have direct and indirect extracellular antimicrobial activity against filarial parasites, fungi, gram-negative bacteria, and gram-positive bacteria [13, 21–23]. Protein levels of S100A12 have been previously reported in patients with leprosy [21], however, the role of S100A12 in macrophage antimicrobial activity against intracellular pathogens, including mycobacteria, has not been investigated. Nevertheless, little is known about the role of S100A12 in infectious disease, since the gene is present in humans but not mice [24]. Studies of inflammatory disease suggest that S100A12, like many antimicrobial proteins, has pro-inflammatory activity [25]. Here, we hypothesized that S100A12 via its antimicrobial activity contributes to host defense against infection by mycobacteria in humans.

**Fig 3:**
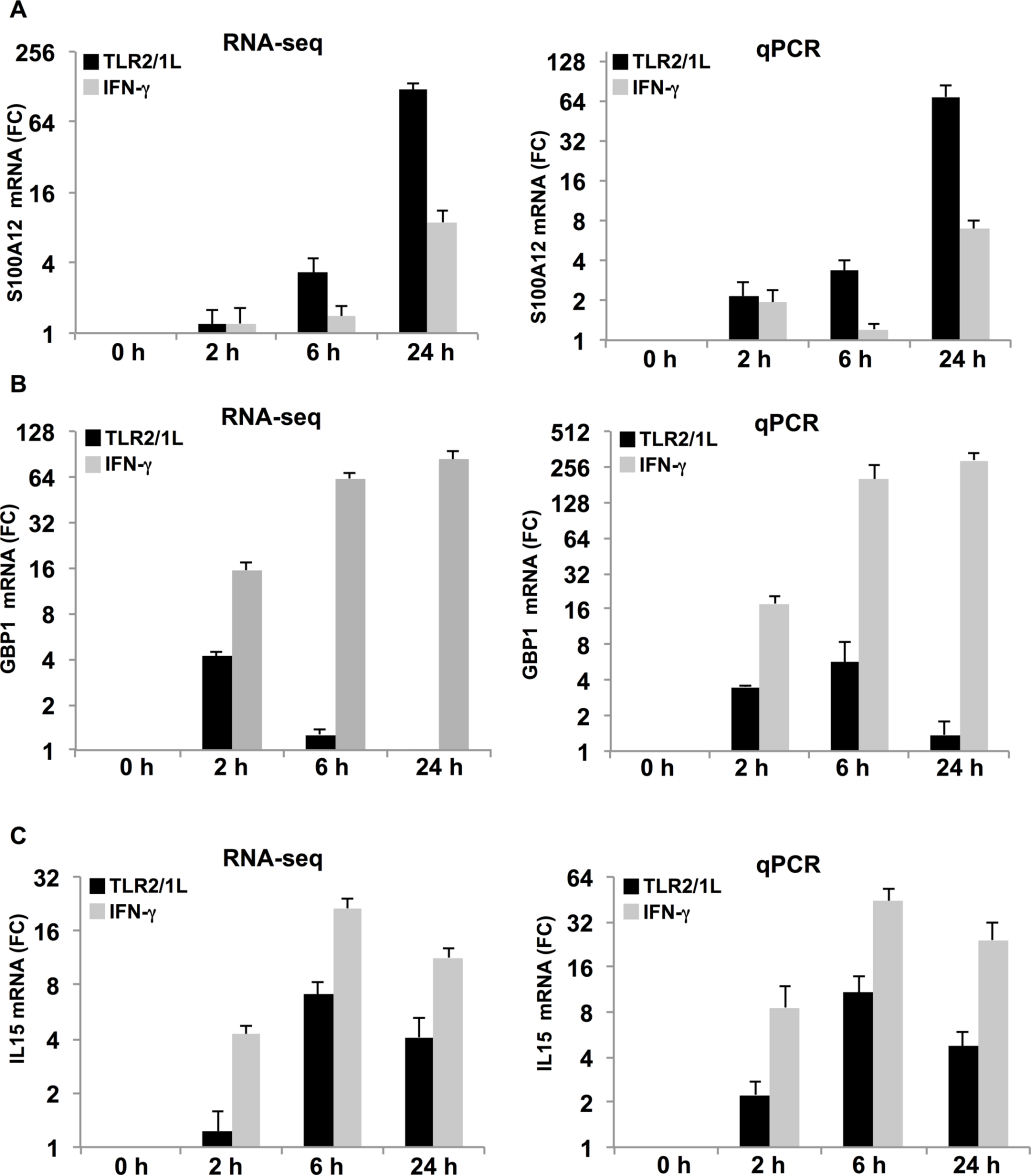
RNAseq gene expression is validated by qPCR. MDMs were stimulated with TLR2/1L (black bars) or IFN-γ (gray bars) for 2, 6, and 24 hours and **(A)** S100A12, **(B)** GBP1, and **(C)** IL15 expression is assessed by RNAseq (left) and quantitative PCR (qPCR) (right). RNAseq results are represented as FC determined by normalized counts in stimulated MDMs versus counts in media control for each time point. Quantitative PCR (qPCR) results were determined by calculating relative arbitrary units using ΔΔCT analysis and normalizing to housekeeping gene. Data are represented as mean FC ± SEM, *n* = 5.

To validate the RNAseq data, the mRNA levels of S100A12 were measured by qPCR in TLR2/1L and IFN-γ activated MDMs (Fig 3), in comparison to GBP1. IL15 served as a control, since we have previously found it to be induced by both TLR2/1L and IFN-γ [7, 26]. Similar to the RNAseq data, S100A12 was more highly expressed following TLR2/1L (FC = 68 at 24 hrs; *P* = 0.001) treatment of MDMs (Fig 3A), although it was also induced by IFN-γ (FC = 7 at 24 hrs; *P* = 0.0002); whereas GBP1 was more highly induced by IFN-γ treatment (FC = 292 at 24 hrs; *P* = 0.0005) (Fig 3B), although also induced by TLR2/1L (FC = 3.5 at 2 hrs, *P* = 2.8 x 10^−7^). As expected, IL-15 mRNA induction was similar for both stimuli (Fig 3C). In addition, a dose titration of TLR2/1L and IFN-γ was performed to ensure that an optimal dose was used for macrophage activation in the induction of S100A12 (S2 Fig). In contrast to the Th1 cytokine, IFN-γ, we also wanted to assess whether a Th2 cytokine, such as IL-4, induces S100A12. MDMs stimulated with IL-4 induced relatively lower levels of S100A12 mRNA (FC = 1.5), as compared to TLR2/1 or IFN-γ treatment in the same donors (S3 Fig). Based on our data, cytokine profiles for TLR2/1L versus IFN-γ stimulated macrophages reveal that TLR2/1L induces pro-inflammatory cytokines such as IL1B (FC = 84), TNF (FC = 21), and IL6 (FC = 1168), which are known to also induce S100A12 expression [27, 28], whereas IFN-γ did not. Therefore, the differential ability of TLR2/1L to induce these pro-inflammatory cytokines may contribute to the greater induction of S100A12.

One antimicrobal gene known to be induced by TLR2/1L and IFN-γ is CAMP, which encodes the antimicrobial peptide cathelicidin. As expected, cathelicidin mRNA was induced by both TLR2/1L and IFN-γ in MDMs (S4 Fig). Cathelicidin mRNA has been previously shown to be induced by treatment of macrophages with the bioactive vitamin D metabolite, 1,25D3 [6, 29]. But unlike cathelicidin mRNA, 1,25D3 treatment was not sufficient to induce S100A12 mRNA in MDMs (S5 Fig), suggesting S100A12 is not likely to be part of the vitamin D antimicrobial pathway. In summary, these data indicate that S100A12 is part of a common set of antimicrobial genes induced in TLR2/1L and IFN-γ activated human macrophages.

### TLR2/1L induced protein expression of S100A12

**Fig 4:**
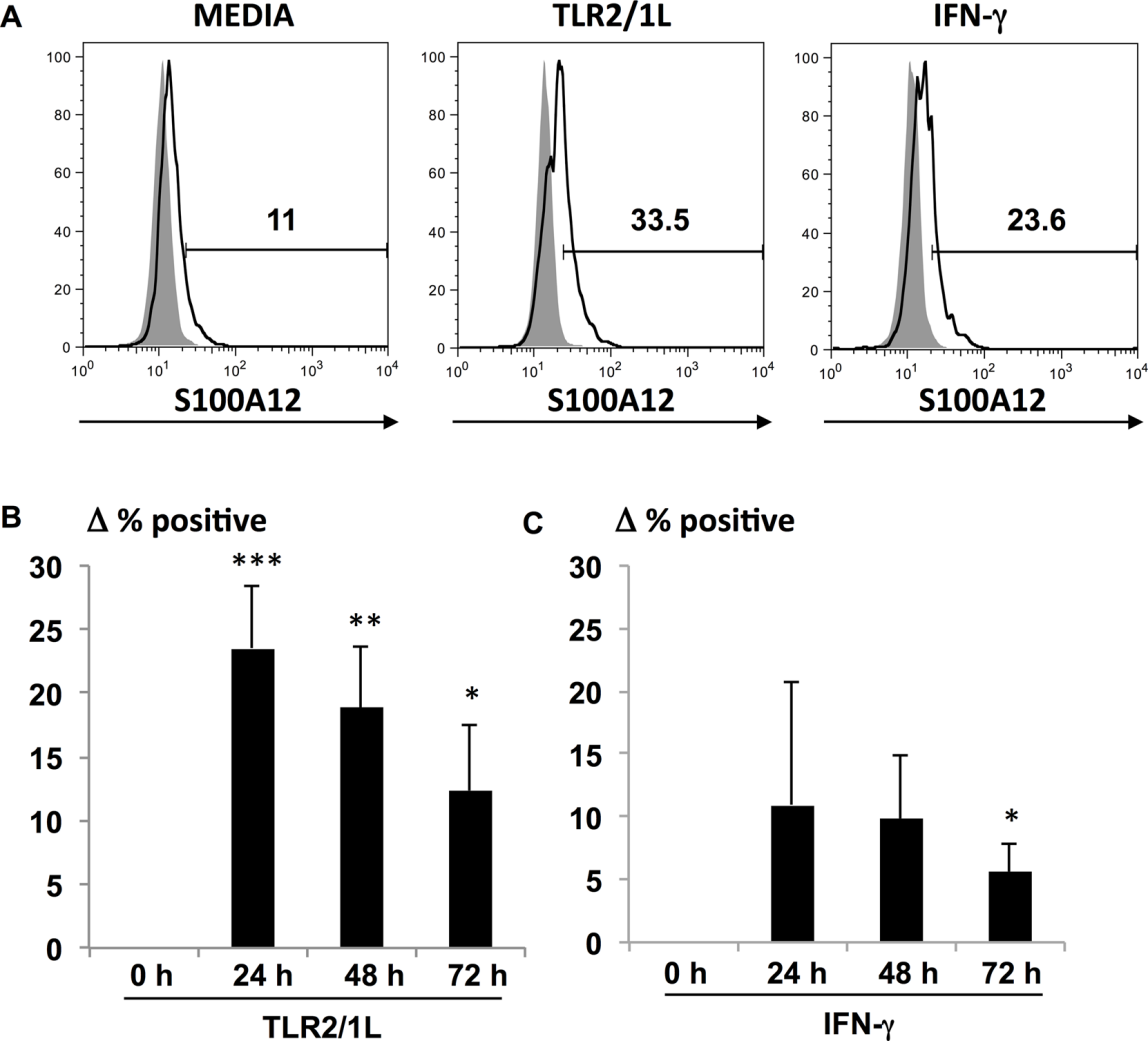
TLR2/1L and IFN-γ inducible S100A12 protein expression. Intracellular S100A12 protein expression following stimulation of MDMs with TLR2/1L or IFN-γ for 24, 48, and 72 hours. **(A)** Representative histogram at 48 hours from flow cytometry showing percent of S100A12 positive cell population (white) with respect to isotype control (gray) in media, TLR2/1L and IFN-γ treated MDMs. Change in percent S100A12 positive cells in **(B)** TLR2/1L (*n* = 9) and **(C)** IFN-γ (*n* = 5) stimulated conditions compared to media control at each time point. Data are represented as mean ± SEM, *P *≤*0.05, **P *≤*0.01.

S100A12 is predominately expressed by myeloid cells. Intracellular expression of S100A12 is predominately detected in the cytosol. The induction of S100A12 intracellular protein in MDMs after stimulation with TLR2/1L or IFN-γ was measured by flow cytometry at 24, 48, and 72 hours, examining the percentage of positive cells compared to media control (Fig 4A). Because the basal expression of S100A12 in untreated cells varied in each donor, we calculated the change in the percentage of positive cells at each time point. The percentage of S100A12 positive cells was significantly greater in cells stimulated with TLR2/1L as compared to media control (Fig 4B), with the greatest percentage being 24% at 24 hours (P = 0.0001). Although the IFN-γ induction of S100A12 positive cells was more variable among the donors (Fig 4C), there is a low but significant increase at 72 hours (P = 0.045). These data show that TLR2/1L and IFN-γ induction of S100A12 mRNA corresponds to protein expression in human macrophages.

### Presence of S100A12 in leprosy lesions

**Fig 5:**
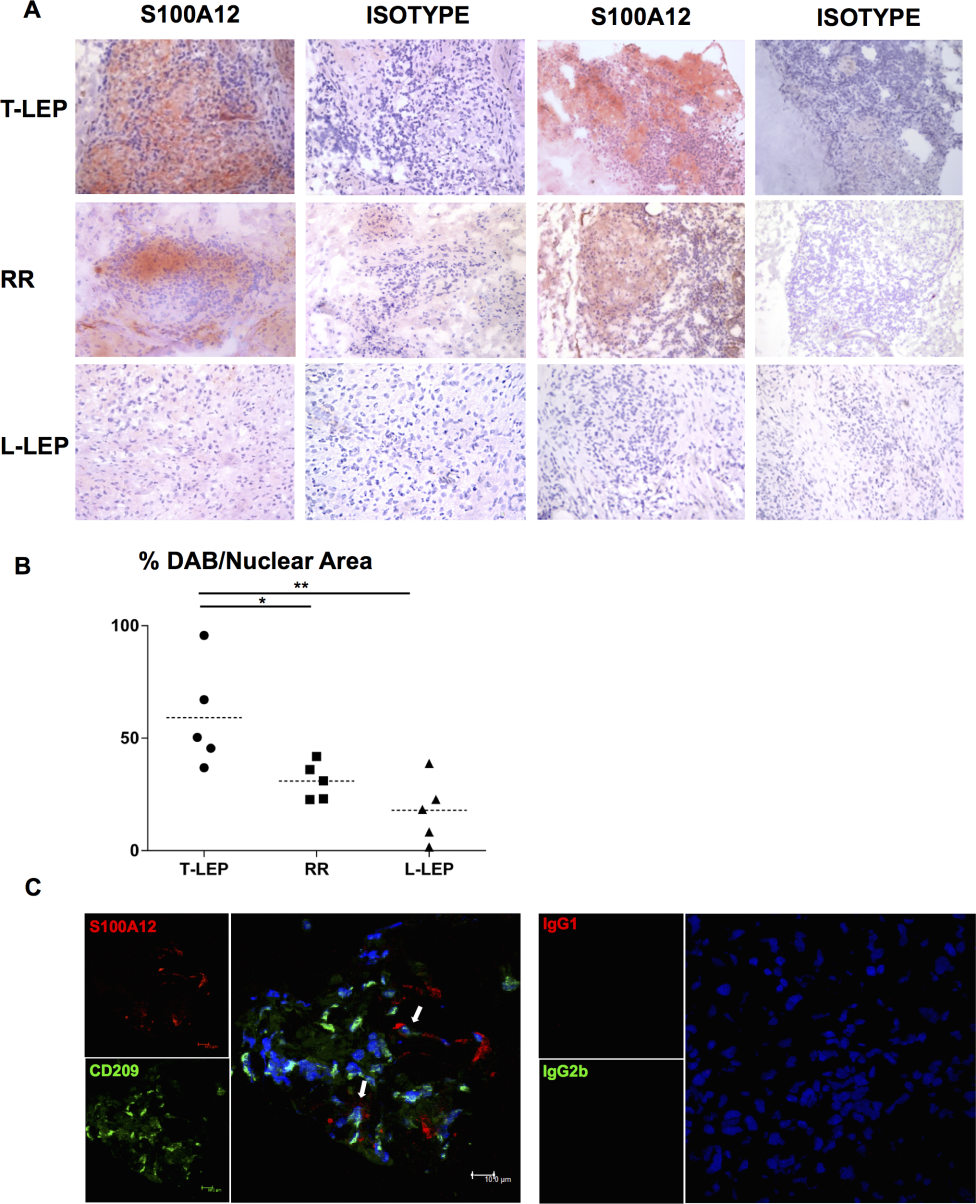
Expression of S100A12 in skin lesions from leprosy patients. **(A)** S100A12 labeling of leprosy skin biopsy specimens by immunoperoxidase. Two representative images are shown for each condition: T-lep, RR, and L-lep, as well as an isotype control for each representative staining. Original magnification: x20. **(B)** The percentage of diaminobenzidine (DAB)-stained area per nuclear area was calculated for each photomicrograph using ImmunoRatio [30]. Each dot represents the %DAB-stained area/nuclear area for each individual photomicrograph. Horizontal dotted line represents mean %DAB per nuclear area for n = 5. One-Way ANOVA analysis was performed (*P* = 0.006) using GraphPad Prism software and post hoc analysis (Turkey test) is indicated by the asterisk (*P<0.05 and ** *P*<0.01). **(C)** Co-expression of S100A12 (red) and CD209 (green) in skin lesion from RR patient. Nucleus was stained with DAPI (blue). Arrows indicate areas of co-expression of S100A12 and CD209; scale bar = 10μm. Original magnification: x40.

To assess the relevance of S100A12 expression at the site of disease in leprosy, S100A12 protein expression was examined in skin lesions from various disease conditions using an anti-S100A12 monoclonal antibody with immunoperoxidase labeling. S100A12 protein was strongly expressed in lesions from T-lep and RR patients when compared to L-lep patients (Fig 5A). Quantification of S100A12 expression in the stained tissues using ImmunoRatio [30] showed T-lep biopsy samples have significantly higher expression of S100A12 compared to L-lep samples, and expression in RR, patients upgrading from the lepromatous to the tuberculoid part of the spectrum, was in between (Fig 5B). This suggests that the expression of S100A12 protein was greatest at the site of disease in patients that are able to limit the infection. Immunofluorescence of lesions from RR patients showed that S100A12 expression can be found at the same site as expression of CD209, a macrophage marker, suggesting macrophages as a potential source of S100A12 production in the lesions or recruitment of S100A12 to the site of disease (Fig 5C). In contrast to our results, S100A12 has been previously reported to be equally present in lesions from patients with the different clinical forms of leprosy [21]. However, this study utilized a polyclonal antibody to measure the expression of S100A12 within lesions, which was produced using Freund’s adjuvant, whereas our studies utilized a monoclonal antibody. Freund’s adjuvant is composed of mycobacterial cell walls, such that in addition to binding S100A12, mycobacterial products would also be detected in those lesions containing bacilli, such as L-lep lesions.

### S100A12 is antimicrobial against mycobacteria

**Fig 6:**
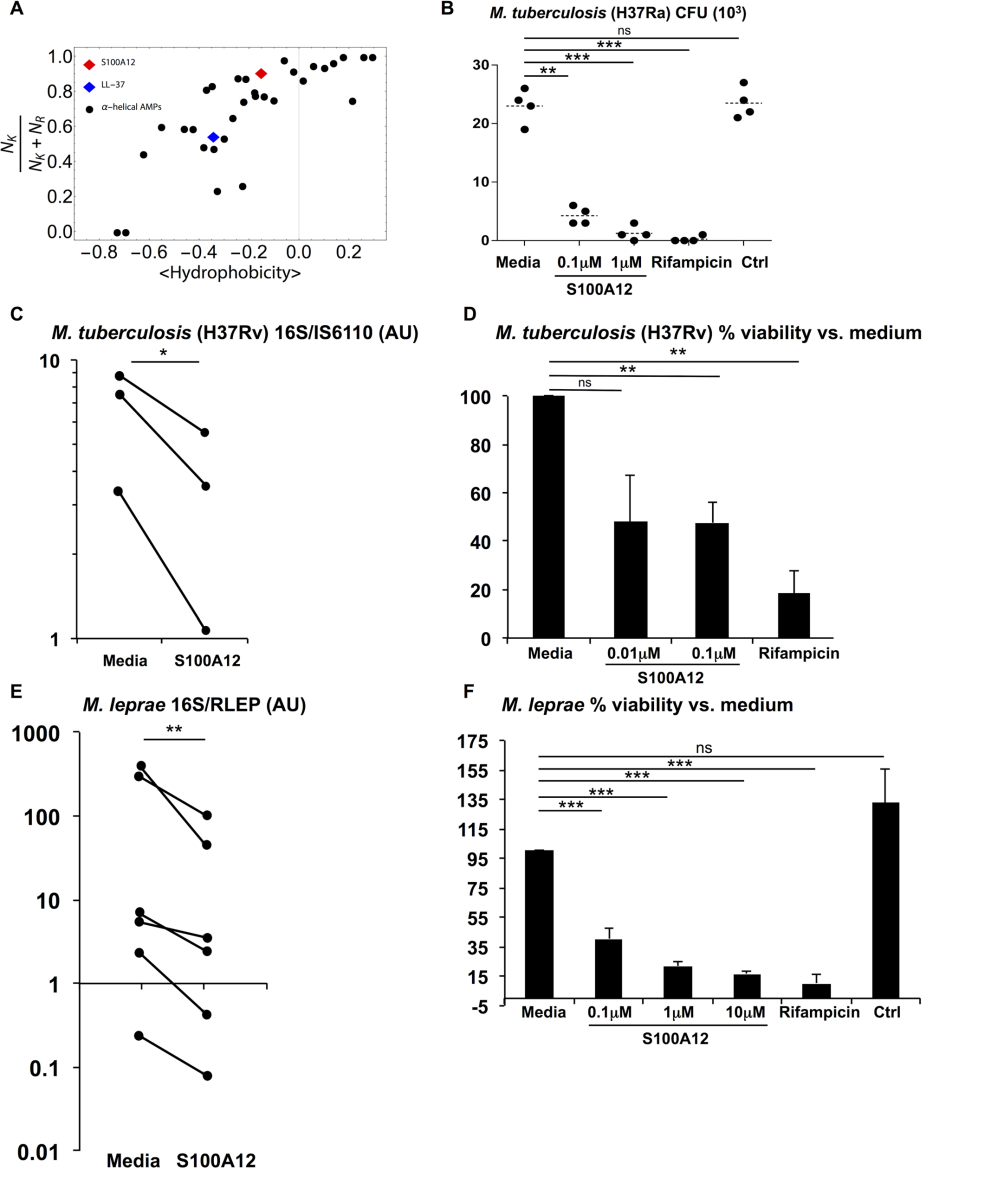
Antimicrobial activity of S100A12 against mycobacteria. **(A)** A plot of (N_K_/(N_K_ + N_R_)) vs. mean hydrophobicity for all known α-helical antimicrobial peptides in the antimicrobial peptide database (http://aps.unmc.edu/AP/main.php). The black circles represent peptides that meet amino acid criterion for membrane curvature generation and exhibit membrane permeating antimicrobial activity. N_K_ and N_R_ are numbers of lysines (K) and arginines (R) on the peptide or protein. Cathelicidin (LL-37) is represented by the blue diamond and S100A12 by the red diamond. **(B)** *M*. *tuberculosis* H37Ra was incubated in the presence of S100A12 protein at the indicated concentrations for 3 days (n = 4). Rifampicin was used as a positive control (20 mg/ml) and IFN-γ protein was used as a negative control. Bacteria were then quantified by CFU assay (n = 4). Each dot represents a replicate sample for each condition indicated (x-axis). **(C)** *M*. *tuberculosis* H37Rv was cultured in the presence of S100A12 protein (0.1μM) or media control for 3 days. Arbitrary units (AU) as determined by the ratio of RNA (16S) to DNA (IS6110) as a measure of viability are shown for H37Rv in media versus S100A12 (n = 3). **(D)** Data from B is represented in % viability relative to media control for in addition to rifampicin control (n = 3). **(E)** *M*. *leprae* was maintained in 7H9 media with S100A12. Shown in AU is the ratio of *M*. *leprae* RNA (16S) to DNA (RLEP) in media control versus S100A12 (0.1μM). Each dot represents a replicate sample for each condition, with a line connecting the AU of each replicate (n = 6). Since there is a large variation in absolute values and ratios, the P-value was determined based on log-transformed values, not log transformed ratios are shown here. **(F)** Data from D is represented as mean % viability, which was calculated with AU relative to media control. In addition, two other concentrations of S100A12 (1μM, n = 7 and 10μM, n = 3), positive control rifampicin (n = 9), and negative control protein (n = 7) are included. Data are represented as mean % viability ± SEM. *P *≤*0.05, **P *≤*0.01, ***P *≤*0.001; ns, non-significant.

S100A12 is a complex multi-⍺-helical protein, with both cationic and amphipathic regions, classic characteristics of antimicrobial peptides [31]. The ability of antimicrobial peptides to alter bacterial membranes requires the generation of negative Gaussian curvature in membranes [32, 33]. This requirement places significant constraints on the amino acid compositions of antimicrobial peptides, such that in order to induce the required curvature, antimicrobial peptides have a characteristic balance between the arginine, lysine, and hydrophobic content. In comparing the composition of S100A12 with the amino acid compositional preferences of known antimicrobial peptides, we find that S100A12 is similar to other antimicrobial peptides. S100A12 is rich in cationic residues (10 lysines, 1 arginine, 6 histidines) as well as hydrophobic amino acids. Quantitative amino acid composition analysis demonstrated that S100A12 follows the same sequence trend of other known α-helical antimicrobial peptides that meet the criterion for negative Gaussian curvature generation and also have membrane permeating antimicrobial activity, such as cathelicidin (LL-37) (Fig 6A).

Next, we examined the ability of S100A12 to exhibit direct antimicrobial activity against *M*. *tuberculosis* and *M*. *leprae* in axenic culture. Bacteria were incubated with recombinant S100A12 protein at several concentrations, while rifampicin served as a positive control. Bacterial viability was assessed after three days in the presence of S100A12 protein versus media control. In the presence of S100A12, the mean colony forming units (CFU) for avirulent *M*. *tuberculosis* (H37Ra) were decreased compared to the media alone (CFU = 23 x10^3^) at two concentrations of S100A12 (CFU = 4x10^3^ at 0.1μM, *P* = 0.003 and CFU = 1x10^3^ at 1μM, *P =* 0.0009) (Fig 6B). S100A12 also showed antimicrobial activity against virulent *M*. *tuberculosis* (H37Rv). Viability of H37Rv was determined by using a previously validated qPCR-based method measuring the ratio of 16S RNA to IS6110 DNA [10, 34] (Fig 6C). The mean percent viability relative to media alone was also determined, showing a significant decrease in the presence of S100A12 (47% viability at 0.1μM, *P =* 0.004) (Fig 6D). Similarly, we also used the ratio of *M*. *leprae* 16S rRNA to repetitive element DNA RLEP, as previously reported, to determine bacterial viability [35, 36]. Although *M*. *leprae* does not grow in culture, we studied the effects of S100A12 in axenic culture, determining whether it further reduced bacterial viability. In the presence of S100A12, we observed a decrease in *M*. *leprae* viability for each replicate (Fig 6E) and in a dose dependent manner (Fig 6F). The antimicrobial activity is consistent with the structure characteristics of S100A12, which are typical of antimicrobial peptides.

### TLR2/1L and IFN-γ inducible antimicrobial activity in human macrophages

**Fig 7:**
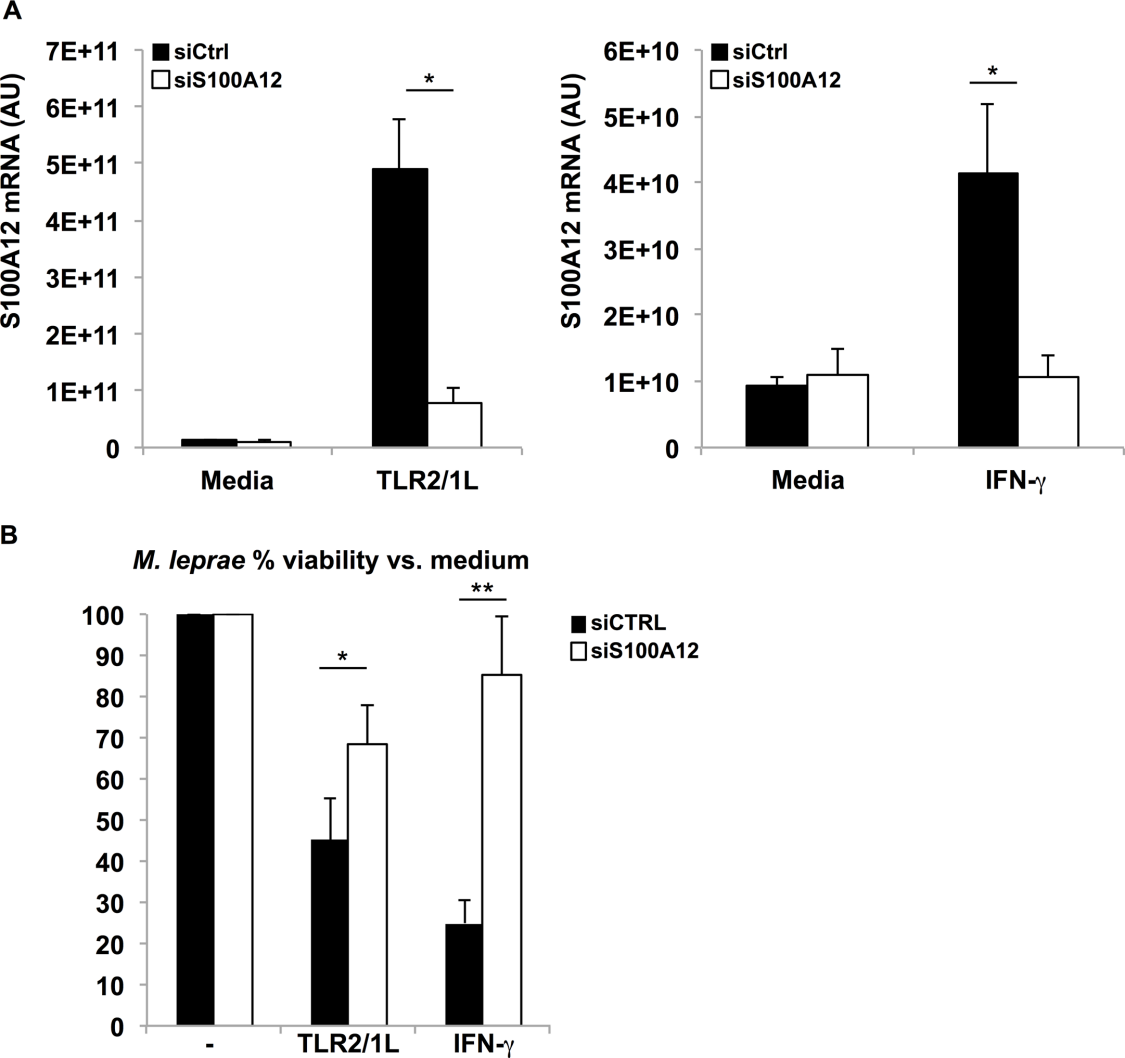
S100A12 plays a role in macrophage antimicrobial activity against *M*. *leprae*. **(A)** MDMs were transfected with siRNA targeting S100A12 (siS100A12) or non-targeting siRNA (siCtrl). qPCR results displaying arbitrary unit (AU) of S100A12 mRNA 24 hours after stimulation with TLR2/1L (*n* = 9) or IFN-γ (*n* = 5). **(B)** MDMs were infected with *M*. *leprae* and stimulated with TLR2/1L (*n* = 9) and IFN-γ (*n* = 8). *M*. *leprae* viability was determined by measuring RNA (16S) and DNA element (RLEP) and determining the % viability relative to respective media control. Data are represented as mean % viability ± SEM, *P *≤*0.05, **P *≤*0.01, ***P *≤*0.001.

To determine whether S100A12 is involved in the TLR2/1L and IFN-γ inducible antimicrobial activity against *M*. *leprae* in macrophages, S100A12 was knocked down in primary human macrophages with a pool of small interfering RNAs (siRNA) targeting S100A12 (siS100A12). S100A12 mRNA expression was inhibited by 84% in TLR2/1L stimulated MDMs and 74% in IFN-γ stimulated MDMs as measured by qPCR in siS100A12 treated cells versus cells treated with non-targeting siRNA (siCtrl) (Fig 7A). Control gene mRNA levels were unaffected by siRNA treatment (S6 Fig). For antimicrobial assays, S100A12 was knocked down in MDMs by siRNA, cells were then treated with TLR2/1L or IFN-γ, followed by overnight infection with *M*. *leprae* at an MOI of 5. After infection, MDMs were stimulated with TLR2/1L or IFN-γ and bacterial viability relative to respective media control was determined after three days of treatment (Fig 7B). The percent of bacterial viability in TLR2/1L and IFN-γ stimulated macrophages was higher in siS100A12 treated cells (TLR2/1L = 68% and IFN-γ = 85%) versus siCtrl (TLR2/1L = 68% vs. 45%, *P* = 0.027 and IFN-γ = 85% vs. 24%, *P* = 0.003) indicating less killing of *M*. *leprae* in the absence of S100A12. These data suggest that S100A12 plays an important role in the TLR2/1L and IFN-γ inducible host defense against *M*. *leprae* in infected macrophages.

## Discussion

The innate and adaptive immune systems each contribute to host defense against intracellular pathogens by triggering macrophage antimicrobial mechanisms. By defining TLR2/1L (innate) and IFN-γ (adaptive) inducible gene networks in human macrophages, we identified a common set of genes with known antimicrobial function. The mRNA and protein encoded by one such gene, S100A12, was induced in macrophages by both TLR2/1L and IFN-γ. Given that S100A12 is present in humans and not mice [24], little is known about its role in human infectious disease, leading us to hypothesize that S100A12 contributes to host defense against mycobacterial infection. By studying the human disease leprosy as a model, we determined that S100A12 was more highly expressed in skin lesions from leprosy patients with the self-limited T-lep vs. the progressive L-lep form, indicating that expression of S100A12 at the site of infection correlates with the ability to contain the infection. Finally, S100A12 was sufficient to kill *M*. *leprae*, as well as *M*. *tuberculosis*, and was required for TLR2/1L and IFN-γ induced antimicrobial activity in *M*. *leprae*-infected macrophages. Together, these data demonstrate that the innate and adaptive immune response induce S100A12 in macrophages as a common antimicrobial mechanism to combat mycobacterial infection.

Previously, S100A12 was shown to have direct antifilarial activity against the nematode *Brugia malayi* in axenic culture [13] and indirect antimicrobial activity against Gram-negative bacterium *Helicobacter pylori* [22], *Candida spp*, and gram-positive bacterium *Listeria monocytogenes* [23]. But to our knowledge, there is little information on the role of S100A12 mediating an antimicrobial activity against a microbial pathogen in human macrophages. Our data indicate S100A12 exhibits antibacterial activity against mycobacteria in axenic culture and also contributes to the innate and adaptive inducible antimicrobial response in human macrophages.

Similar to other antimicrobial proteins, S100A12 has also been shown to modulate host immune responses. Through the activation of RAGE and TLR4, S100A12 activates MAP kinase and NF-κB pathways [27, 37]. As a result, S100A12 can induce cytokine secretion and increase expression of adhesion molecules in activated cells [38]. Other immunomodulatory activities of S100A12 include the induction of cell migration and chemotaxis of monocytes [39]. In our analysis, S100A12 was linked to gene ontology terms “chemotaxis” and “inflammatory response”, in addition to “defense response” and “killing of cells of other organisms”. High levels of S100A12 protein have been associated with several inflammatory diseases such as rheumatoid arthritis, inflammatory bowel disease, and psoriasis [40–42]. As a result, many studies suggest levels of S100A12 can be representative of disease state and can serve as a clinical biomarker for inflammation. Although, S100A12 can be detected systemically as well as in inflammatory compartments and tissues, the specific role and function of S100A12 in these disease states is not well defined. The expression of S100A12 in patients with RR, able to restrict the infection but with concomitant nerve damage, suggests that like many antimicrobial peptides, the antimicrobial and inflammatory functions of these molecules are linked [43, 44].

In addition to S100A12, we identified 15 additional antimicrobial genes that were induced by both stimuli, including calgranulins S100A8 and S100A9, chemokine CXCL6, and guanylate binding proteins (GBPs). S100A12 is a member of the S100 calgranulins, a subgroup of myeloid-related proteins, which also includes S100A8 and S100A9. Calgranulins are proteins involved in intracellular and extracellular activities during inflammation and infection, serving as antimicrobial proteins as well as damage-associated molecular pattern molecules [45]. S100A8/A9 antimicrobial heterocomplex has been reported to inhibit the growth of several pathogens, but stimulate the growth of *M*. *tuberculosis* in liquid media [46]. It is possible that the effects of S100A8/A9 may differ in infected cells; therefore further studies in human macrophages are needed. In addition, GBPs play a role in IFN-γ inducible anti-mycobacterial activity in murine macrophages [15], however the role of GBPs in TLR2/1L or IFN-γ inducible anti-myocbacterial activity in infected human macropahges still needs to be confirmed. Our data also indicated a divergent set of antimicrobial genes that were significantly induced by either TLR2/1L or IFN-γ but not both. Only IFN-γ induced DEFB1, CXCL10, and IL32. IL-32 is part of the IFN-γ inducible vitamin D-dependent antimicrobial pathway, leading to the induction of CAMP and DEFB4 and the killing of *M*. *tuberculosis* [10]. However, IL-32 is not induced by TLR2/1L, therefore represents an IFN-γ mediated antimicrobial pathway. On the other hand, TLR2/1L induced additional antimicrobial S100 genes, such as S100A7 and S100A7A, suggesting they could be involved in a TLR2/1L divergent host defense against mycobacteria. S100A7 and S100A7A are constitutively expressed in the skin and can be present at high levels during disease or inflammation, such as, for example, psoriasis [47, 48]. Together, these genes complement the multiple effector mechanisms contributing to antimicrobial activity in infected macrophages. However, the role of these proteins encoded by S100 genes as well as other antimicrobial genes identified here remains to be determined in host defense responses against mycobacteria. Nevertheless, these data indicate that the innate and adaptive immune systems converge on a matrix of antimicrobial proteins as part of a common “host defense” pathway.

TLR2/1L and IFN-γ have been previously shown to converge on a common antimicrobial pathway with the vitamin D-dependent induction of the antimicrobial genes CAMP and DEFB4. LL-37 and β-defensin 2, the peptides encoded by CAMP and DEFB4, were both sufficient to kill mycobacteria, and individually were required for TLR2/1 mediated-antimicrobial activity [29, 36]. Although CAMP was inducible by 1,25D3, we determined that 1,25D3 was not sufficient to induce S100A12 mRNA in MDMs. The fact that S100A12 does not have a positive vitamin D receptor element in its promoter, suggests that vitamin D is not required for the induction of S100A12. Therefore, both vitamin D dependent and independent antimicrobial pathways contribute to host defense against mycobacteria in humans.

In summary, our data demonstrate the role of S100A12 in the antimicrobial activity against mycobacteria and the existence of a convergent and divergent host defense gene network. The induction of TLR2/1L and IFN-γ common antimicrobial network, which includes S100A12, provides evidence for the existence of multiple antimicrobial effector mechanisms in activated macrophages that act to eliminate pathogens. In addition to defense response, TLR2/1 and IFN-γ trigger a network of other immunomodulatory functions such as T cell activation and antigen presentation. Understanding the antimicrobial effector mechanisms is important for developing host directed therapies or modulating the host immune response in order to enhance the immune system to effectively combat intracellular pathogens such as mycobacteria.

## Methods

### Antibodies and cytokines

Human recombinant S100A12 protein (R&D systems) was used for antimicrobial assays in axenic cultures. Human recombinant M-CSF (R&D systems) was used to differentiate monocytes into monocyte-derived macrophages (MDMs). TLR2/1 ligand (TLR2/1L) used for stimulating MDMs was a synthetic 19kDa lipoprotein derived from mycobacteria (EMC Microcollections). Recombinant human IFN-γ (BD Biosciences) and IL-4 was also used for stimulating MDMs. Antibody used for intracellular S100A12 protein detection was mouse monoclonal human anti-S100A12 clone 1D1 (Novus Biologicals) and matched isotype IgG1 was used as a control. Anti-CD209 clone DCN46 and matched isotype IgG2b was used for immunofluorescence staining. Isotype-specific, fluorochrome (A488, A568)-labeled goat anti-mouse immunoglobulin antibodies (Molecular Probes) were used as secondary antibodies for immunofluorescence.

### Ethics statement

Human peripheral blood was obtained from healthy donors with informed consent (UCLA Institutional Review Board #11–001274). Written informed consent was provided by all study participants.

### Macrophage cultures

Peripheral blood mononuclear cells (PBMCs) were isolated from peripheral blood using Ficoll-hypaque (GE Healthcare) density gradient. MDMs were generated as previously described [7, 10]. In brief, CD14 positive cells were subsequently isolated from PBMC using CD14 MicroBeads (Miltenyi Biotec) for positive selection according to manufacturer’s suggested protocol. Purity was confirmed by flow cytometry, showing >90% yield for CD14 positive cells. For monocyte differentiation, CD14 positive cells were cultured with M-CSF (50 ng/ml) in RPMI 1640 supplemented with 10% FCS (Omega Scientific) for 4–5 days.

### RNA sequencing

MDMs generated from five healthy donors were stimulated with TLR2/1L (1 μg/ml) or IFN-γ (1.5 ng/ml) in RPMI supplemented with 10% vitamin D sufficient pooled non-heat inactivated human serum. Dose titrations were performed in order determine concentrations for TLR2/1L and IFN-γ by looking at expression of genes of interest and phosphorylation of signaling factors. Concentrations used in the dose titration were chosen based on levels previously reported to induce an antimicrobial response against mycobacteria in monocytes and macrophages [5–7, 36]. Total RNA was harvested using RLT buffer (1% β-mecaptoethanol supplemented) from RNAeasy Micro kit (Qiagen) at 2, 6, and 24 hours. Media control samples were also harvested at each time point. RNA extraction was performed according to manufacturer’s instructions, which included the on-column DNAse treatment step. Extracted RNA was quantified with Quant-iT RiboGreen RNA Assay Kit (Invitrogen) and RNA quality was assessed using the Agilent 2200 Tapestation. Total RNA was subjected to poly-A-selection to purify messenger RNA, then fragmented and converted into double stranded cDNA. Library construction was then done using TruSeq Sample Preparation Kit (Illumina) according to manufacturer’s instructions. This included the ligation of sequencing adapters containing 7 nucleotide indexes for multiplexing. Libraries were quantified using PicoGreen (Invitrogen) and quality was assessed using the Agilent 2200 Tapestation. Library samples were pooled (4 per lane) at equimolar quantities (10uM each library) and sequenced on a HiSeq 2000 sequencer (Illumina) with 100bp single-end protocol.

### Bioinformatics analysis

Sequenced reads were demultiplexed and aligned to the human reference genome hg19 (UCSC) using TopHat (version 2.0.6) and Bowtie2 (version 2.0.2). The HTseq package was then used to assign uniquely mapped reads to exons and genes using the gene annotation file for build hg19 from Ensembl in order to generate raw count data. Once raw count data was generated, data normalization and differential expression analysis using a negative binomial model was performed in the R statistical programming environment using the DESeq (version 1.5) Bioconductor package. *P*-values were adjusted for multiple testing using the Benjamini-Hochberg correction. A cutoff of less than 0.01 for *P*-values and a fold change greater than 2 compared to media control was used to identify significant differentially expressed genes. Hierarchical clustering was performed using the ‘hclust’ command in R.

### Weight Gene Network Correlation Analysis (WGCNA)

WGCNA was performed using the “WGCNA” R package as previously described [9]. Ensembl IDs were filtered first by excluding genes with 0 count across all samples, then by calculating the overall sum of counts across all samples and removing genes in the lowest 40% quantile range. All samples were analyzed simultaneously. The function “blockwiseModules()” was used to construct signed hybrid, weighted correlation networks with a soft thresholding power of 10. Module correlation was generated by coding traits (Media, TLR2/1L, and IFN-γ) as a binary matrix of zeros and ones: each sample had a value of ‘1’ for its corresponding subtype and ‘0’ for all other subtypes. The WGCNA built-in ‘Heatmap’ function was used to display the correlation and significance (p-value) of traits versus modules.

### Functional gene annotation analysis

A curated set of genes with direct and indirect antimicrobial related function, not including ones with specific antiviral function, was generated from the Antimicrobial Peptide Database [49], Ingenuity Knowledge Base (Qiagen), Database for Annotation, Visualization and Integrated Discovery (DAVID), and a literature review.

Gene ontology (GO) enrichment analysis was performed using Cytoscape (version 3.2.0) software with the ClueGO (version 2.1.5) plugin [50]. The GO term database file (received March 20^th^, 2014) was used and the significance of each term was calculated with a right-sided hypergeometric test with Benjamini-Hochberg correction of *P*-values. Significantly overrepresented terms were defined as having a *P*-value less than 0.05, a minimum of 4 genes per term, and at least 6% of the genes from the dataset associated with the term. Functionally similar GO terms were grouped into simplified representative terms. A functional annotation network to represent the genes associated with simplified terms was generated with the interactive visualization platform Gephi (version 0.8.2 beta).

### Real-time quantitative PCR

MDMs were stimulated with TLR2/1L, IFN-γ, 1,25D3 (BioMol), or media control. Total RNA was isolated using Trizol reagent (Invitrogen) and cDNA was prepared as previously described [6]. RT-PCR was performed using KAPA SYBR FAST qPCR Kit (Kapa Biosystems) and normalized to h36b4 and relative arbitrary units were calculated using ΔΔC_T_ analysis [6]. Primer sequences for human S100A12 are: S100A12 Forward, CGGAAGGGGCATTTTGACACC, S100A12 Reverse, CCTTCAGCGCAATGGCTACC. GBP1 Forward, CGACAGGGTCCAGTTGCTGAA, GBP1 Reverse, TTCGTCGTCTCATTTTCGTCTGG. Primer sequences for h36b4, CAMP, S100A8, and IL15 were previously reported [6, 51, 52].

### Flow cytometry

MDMs were stimulated with TLR2/1L or IFN-γ for 24, 48, and 72hrs. Adherent cells were detached using a cell scraper and treated with Cyto Fix/Cyto Perm Solution (BD Biosciences) for fixation and permeabilization. A two-step intracellular flow procedure was performed using anti-S100A12 or isotype control for primary antibody incubation, followed by secondary goat anti-mouse AlexaFlour488 antibody. Samples were processed on an LSRII (BD Biosciences) in the UCLA Jonsson Comprehensive Cancer Center (JCCC) and Center for AIDS Research Flow Cytometry Core Facility.

### Primary macrophage siRNA transfection

MDMs were transfected with SMARTpool Accell siRNA targeting S100A12 (E-012139-00-0005) and control non-targeting pool (D-001910-10-05) (GE Dharmacon). Lipofectamine-siRNA complexes were formed by mixing 1ul of Lipofectamine RNAiMax Transfection Reagent (Invitrogen) and 20pmol of siRNA construct according to manufacturer's instructions. MDMs were seeded in a 24-well plate at 5 x 10^4^ cells, and each was transfected with Lipofectamine RNAiMAX-complexes for 6 hours at 37°C and 5% CO_2_ in RPMI with 10% FCS. Cells were then washed and placed in fresh differentiation culture media (RPMI, 10% FCS with M-CSF) for 24 hours to recover. Transfected MDMs were then stimulated with TLR2/1L or IFN-γ for 24 hours or infected with bacteria as indicated. For antimicrobial assays, a second round of siRNA was given after infection with bacteria.

### Leprosy biopsy specimens, immunoperoxidase labeling, and immunofluorescence

Skin biopsy specimens were collected from untreated patients at the Hansen’s Disease Clinic at Los Angeles Country and University of Southern California Medical Center as well as the Leprosy Clinic at the Oswaldo Cruz Foundation in Brazil. The diagnosis and classification of patients was determined based on clinical and histopathological criteria of Ridley and Jopling. Cryosections of skin lesions from T-lep, L-lep, and RR patients were stained for S100A12. Sections were incubated with normal horse serum followed by incubation with anti-S100A12 antibody or matched isotype control. Following primary antibody incubation, sections were incubated with biotinylated horse anti-mouse IgG and visualized by ABC Elite system (Vector Laboratories) and AEC Peroxidase Substrate Kit (Vector Laboratories). The section was then counterstained with hematoxylin and mounted with crystal mounting medium (Biomeda). No eosin staining was used in this procedure. Expression of S100A12 in photomicrographs was quantified using ImmunoRatio [30], an automated image analysis application which calculates the percent diaminobenzidine (DAB)-stained nuclear area per total area.

Two-color immunofluorescence was performed on cryostat tissue section by incubating with anti-S100A12 antibody (Clone: 1D; IgG1) and anti-CD209 (Clone: DCN46; IgG2b) followed by secondary isotype-specific, flourochrome (A488 and A568)-labeled goat anti-mouse antibodies. Immunofluorescence of skin sections were visualized using Leica SP2 1P-FCS confocal microscope at the Advanced Microscopy/Spectroscopy Laboratory Macro-Scale Imaging Laboratory, California Nanosystems Institute, UCLA.

### Live *M*. *leprae* and *M*. *tuberculosis*

*M*. *leprae* was provided by Dr. James L. Krahenbuhl of the National Hansen's Disease Programs, Health Resources Service Administration, Baton Rouge, LA. The bacteria was grown in the footpad of nu/nu mice and treated with NaOH, as described previously [36]. *M*. *tuberculosis* strains (H37Ra and H37Rv) were cultured as previously described [10, 53]. Frozen stocks were plated on 7H11 agar plates for 3–4 weeks at 37°C and 5%CO_2_. Bacteria were harvested from solid colonies and a single cell suspension was prepared by sonication followed by centrifugation. Experiments with *M*. *tuberculosis* H37Rv were performed in a biosafety level 3 facility at UCLA.

### Composition analysis of S100A12 and α-helical antimicrobial peptides

We sourced 31 α-helical antimicrobial peptides from the antimicrobial peptide database and analyzed them using a procedure previously described [32, 54]. For each peptide sequence, we calculated the average hydrophobicity using the following equation for a given peptide *x*

$${\left\langle hydrophobicity\right\rangle }_{x}\equiv \frac{1}{n}\sum_{i=1}^{n}w_{i}$$

where *n* is the number of amino acids in the peptide, and *w*_i_ is the hydrophobicity of the *i* ^th^ amino acid in the peptide using the Eisenberg consensus hydrophobicity scale [55]. To calculate the lysine fraction, we use the following equation for each peptide *x*:

$$\frac{N_{K}}{N_{K}+N_{R}}\equiv \frac{{\left(number\;of\;K\right)}_{x}}{{\left(number\;of\;K\right)}_{x}+{\left(number\;of\;R\right)}_{x}}$$

We add up the number of lysines (K) and divide it by the total number of lysines (K) and arginines (R) for each peptide. For each peptide, N_K_/(N_K_ + N_R_) vs. <hydrophobicity> was plotted. Human cathelicidin (LL-37) and S100A12 were analyzed and plotted using the same procedures.

### Antimicrobial assays

For direct cell-free antimicrobial killing experiments, bacteria was maintained in 7H9 Middlebrook media in the presence and absence of human S100A12 protein diluted in sodium phosphate (pH 7.4) for 3 days at 33°C 7% CO_2_. Rifampicin (Sigma) was used a positive control for killing and IFN-γ protein was used as a negative control. Macrophage antimicrobial experiments were performed as previously described [5], in brief MDMs were pre-stimulated with TLR2/1L, IFN-γ, or media 6 hours prior to infection with *M*. *leprae*. Infection was done overnight (MOI 5) in RPMI with 10% FCS. After overnight infection, cells were post-stimulated with TLR2/1L, IFN-γ, or media for 3 days in RPMI supplemented with vitamin D sufficient serum. RNA and DNA was isolated using Trizol reagent by using the phenol-chloroform and back-extraction method, respectively (manufacturer’s suggested protocol). Isolated RNA was DNAse (Qiagen) treated and cDNA was subsequently synthesized using the iScript cDNA Synthesis Kit (Bio-Rad).

Bacterial viability was determined by CFU (H37Rv) or by a quantitative PCR (qPCR) based method as previously described [35, 36]. Briefly, the levels of bacterial 16S rRNA and a genomic DNA element, RLEP for M. leprae and IS6110 for *M*. *tuberculosis*, were measured by qPCR. The 16S RNA and DNA element values were determined by using the ΔΔCT analysis, with the DNA value serving as a housekeeping gene. The ratio of RNA to DNA was calculated for each replicate and the percent of bacterial viability was calculated relative to the respective media control. Primers for *M*. *leprae* 16S and RLEP and *M*. *tuberculosis* 16S and IS6110 were previously reported [35, 36, 53].

### Statistical analysis

Results from individual donors are represented as mean ± SEM. Data was analyzed using a Student’s *t* test, with statistical significance at *P* <0.05. Quantitative data from immunohistological staining was analyzed using GraphPad Prism.

## Supporting Information

S1 Table ClueGO analysis of WGCNA modules. (XLSX)

S2 Table ClueGO analysis of TLR2/L and IFN-γ common genes. (XLSX)

S1 Fig Summary of direct antimicrobial activity against a broad-spectrum of pathogens of TLR2/1L and IFN-γ inducible genes. (TIFF)

S2 Fig Dose titration for S100A12 expression in TLR2/1L and IFN-γ stimulated MDMs. MDMs were stimulated with indicated concentrations of TLR2/1L or IFN-γ for 24 hours. S100A12 mRNA levels were measured by qPCR (mean FC ± SEM, *n* = 3).
(TIFF)

S3 Fig TLR2/1L and IFN-γ induction of S100A12 is greater than stimulation with IL-4. MDMs were stimulated with TLR2/1L (1ng/ml), IFN-γ (1.5ng/ml) or IL-4 (20ng/ml) for 24 hours. S100A12 mRNA levels were measured by qPCR (mean FC ± SEM, *n* = 3).
(TIFF)

S4 Fig TLR2/1L and IFN-γ CAMP induction in MDMs. MDMs were stimulated with TLR2/1L or IFN-γ. Cathelicidin mRNA was measured by qPCR. Cathelicidin mRNA expression was not detected by RNAseq but detected by qPCR of the same mRNA samples, indicating a lower senstivity of the RNAseq approach for this particular gene. Max FC from each donor at 2, 6, or 24 hours represented as mean ± SEM, (*n* = 5).
(TIFF)

S5 Fig 1,25D3 alone does not induce S100A12. MDMs treated with 1,25D3 in 10% FCS for 24 hours induced CAMP mRNA, but not S100A12 mRNA as measured by qPCR (mean FC ± SEM, *n* = 4).
(TIFF)

S6 Fig TLR2/1L and IFN-γ induced expression of S100A8 is unchanged by siS100A12 knockdown. MDMs were transfected with siRNA specific for S100A12 (siS100A12) or non-specific (siCtrl) and subsequently treated with TLR2/1L (*n* = 9) or IFN-γ (*n* = 5) for 24 hours. S100A8 mRNA was assessed by qPCR (mean FC ± SEM).
(TIFF)
