# Supplementary figures and images for: S100A12 Is Part of the Antimicrobial Network against Mycobacterium leprae in Human Macrophages
Source: PLoS Pathog. 2016 Jun 29;12(6):e1005705. doi: 10.1371/journal.ppat.1005705 (PMC4927120; doi:10.1371/journal.ppat.1005705)

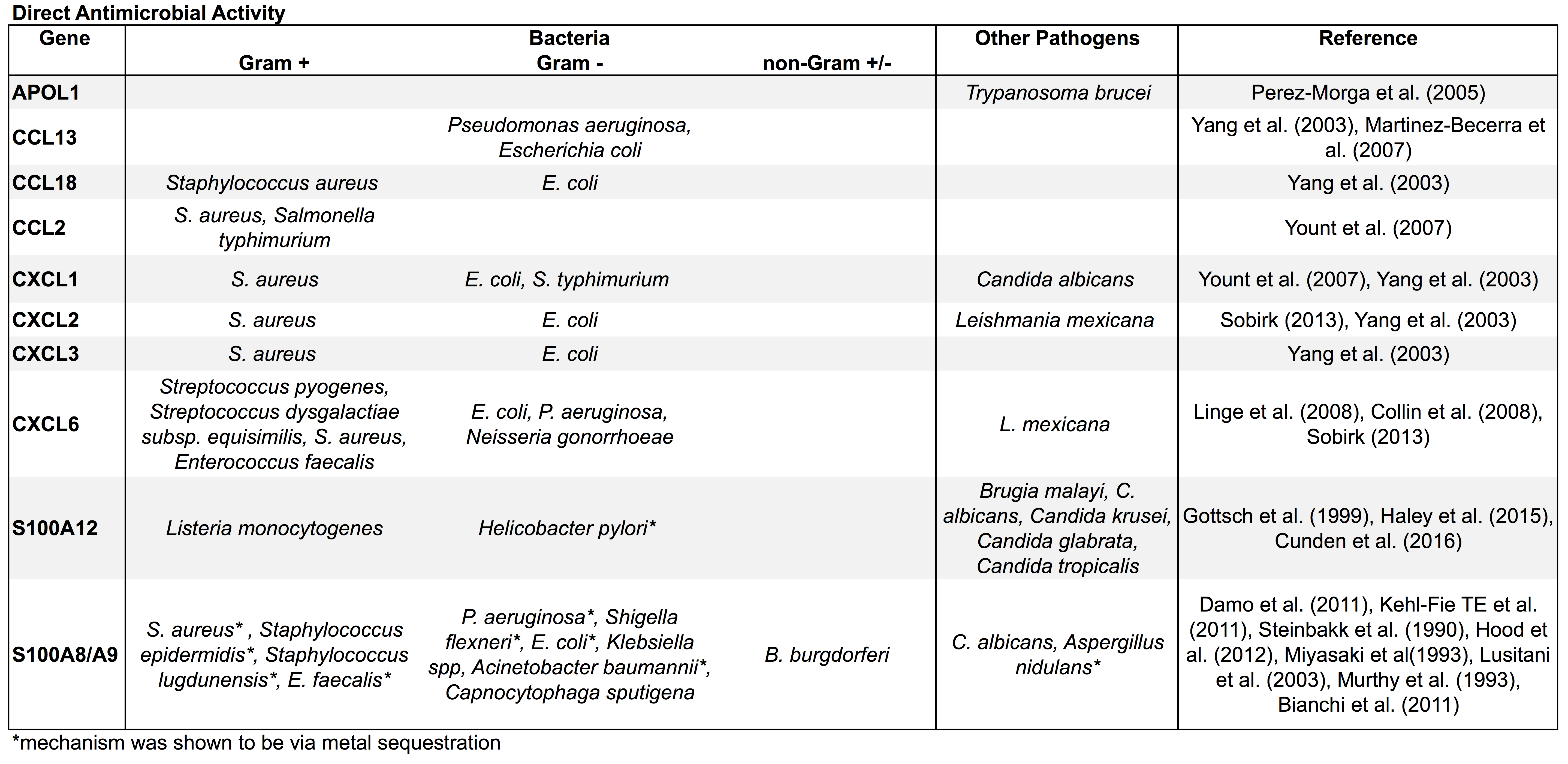

Supplement: S1 Fig — (TIFF) [file ppat.1005705.s003.tiff]

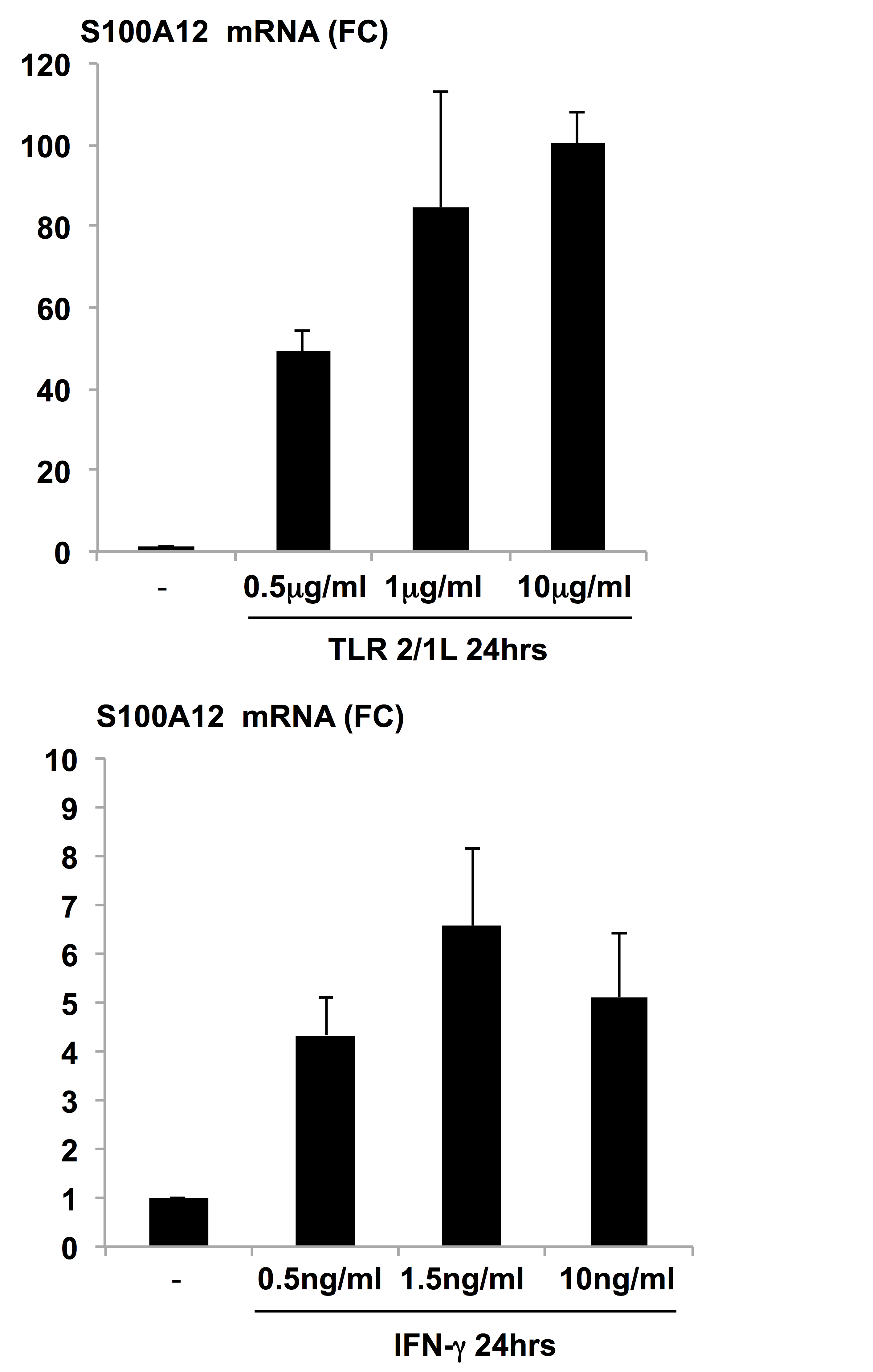

Supplement: S2 Fig — MDMs were stimulated with indicated concentrations of TLR2/1L or IFN-γ for 24 hours. S100A12 mRNA levels were measured by qPCR (mean FC ± SEM, n = 3). (TIFF) [file ppat.1005705.s004.tiff]

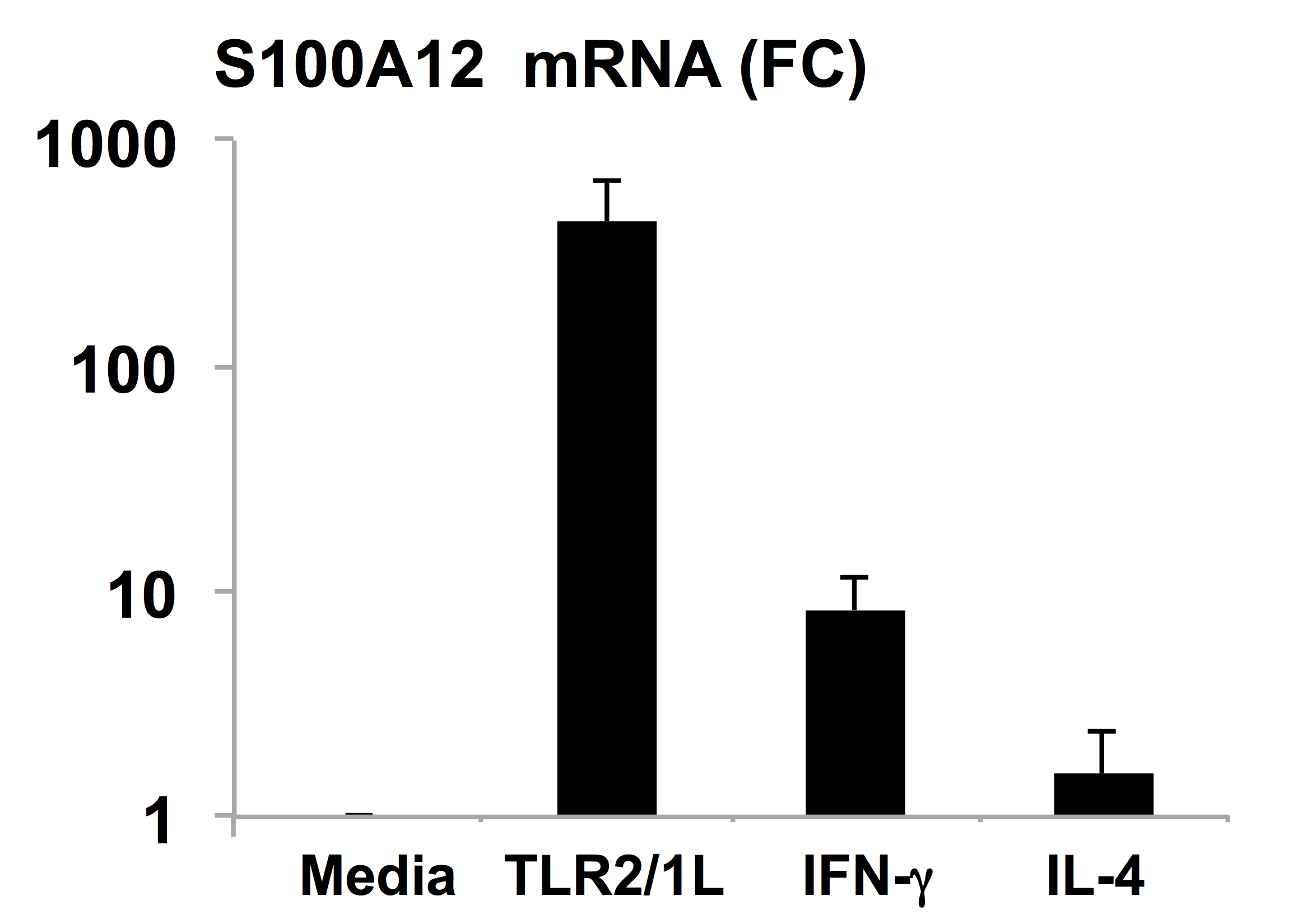

Supplement: S3 Fig — MDMs were stimulated with TLR2/1L (1ng/ml), IFN-γ (1.5ng/ml) or IL-4 (20ng/ml) for 24 hours. S100A12 mRNA levels were measured by qPCR (mean FC ± SEM, n = 3). (TIFF) [file ppat.1005705.s005.tiff]

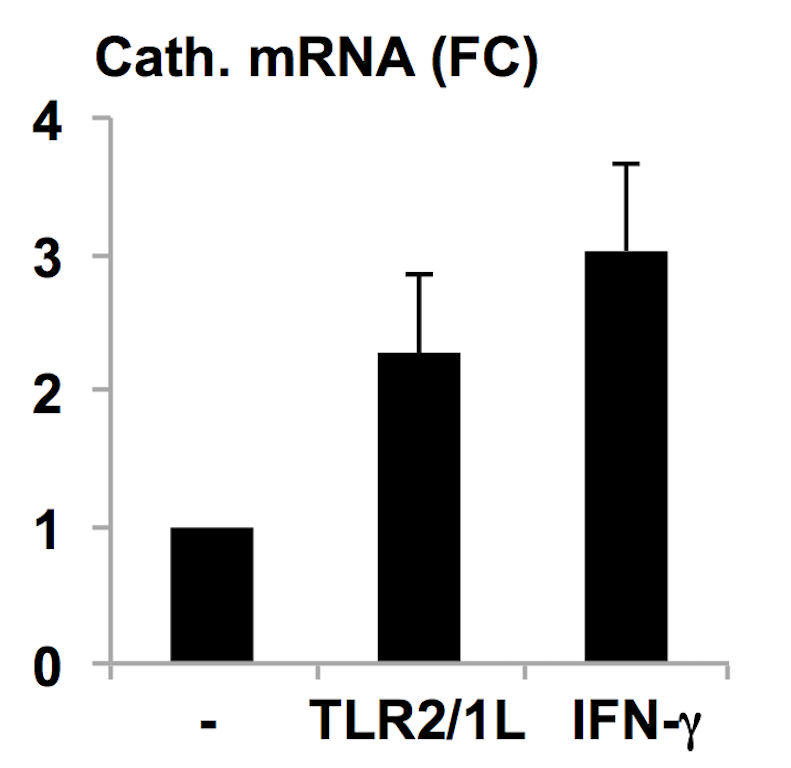

Supplement: S4 Fig — MDMs were stimulated with TLR2/1L or IFN-γ. Cathelicidin mRNA was measured by qPCR. Cathelicidin mRNA expression was not detected by RNAseq but detected by qPCR of the same mRNA samples, indicating a lower senstivity of the RNAseq approach for this particular gene. Max FC from each donor at 2, 6, or 24 hours represented as mean ± SEM, (n = 5). (TIFF) [file ppat.1005705.s006.tiff]

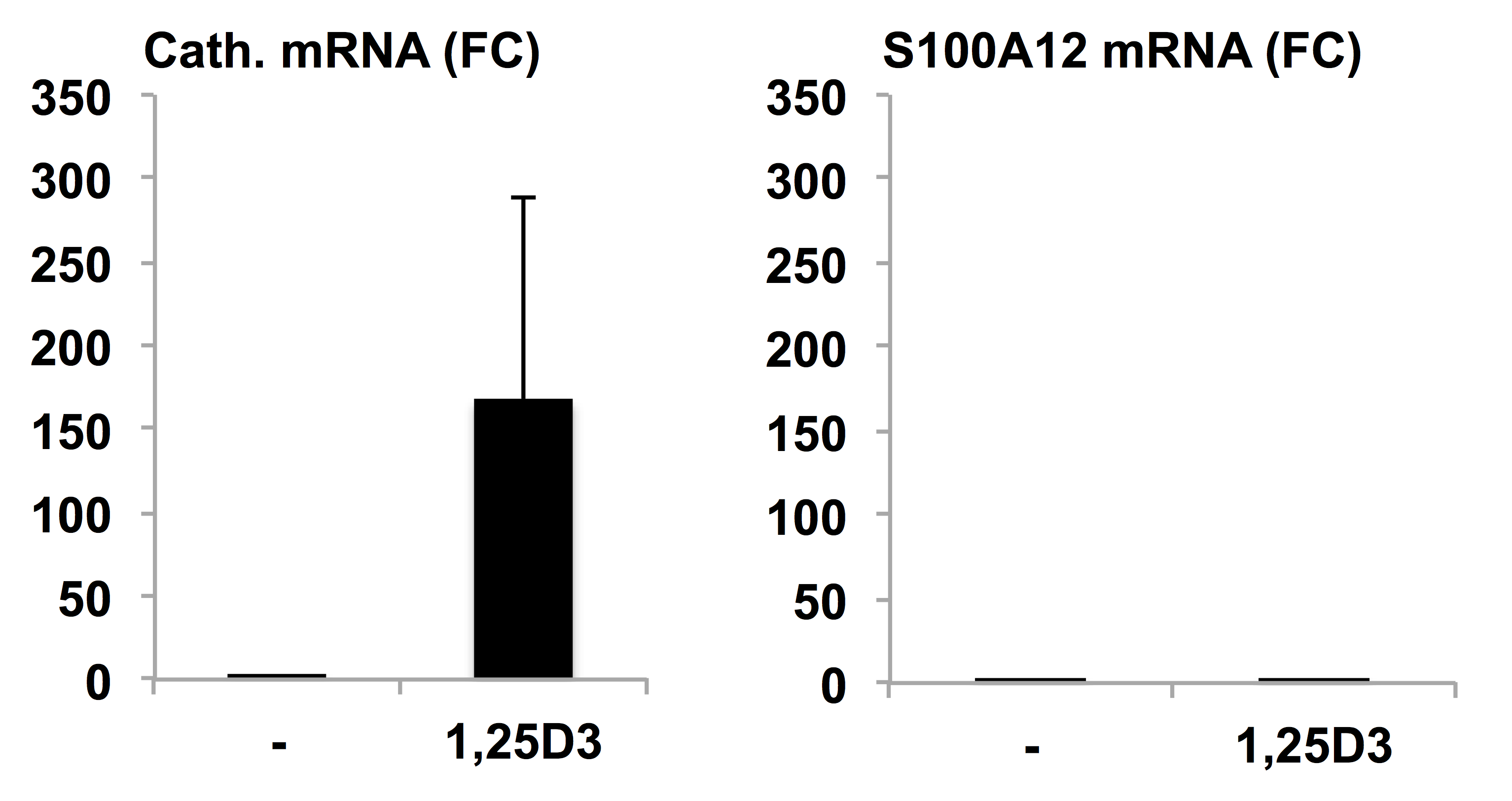

Supplement: S5 Fig — MDMs treated with 1,25D3 in 10% FCS for 24 hours induced CAMP mRNA, but not S100A12 mRNA as measured by qPCR (mean FC ± SEM, n = 4). (TIFF) [file ppat.1005705.s007.tiff]

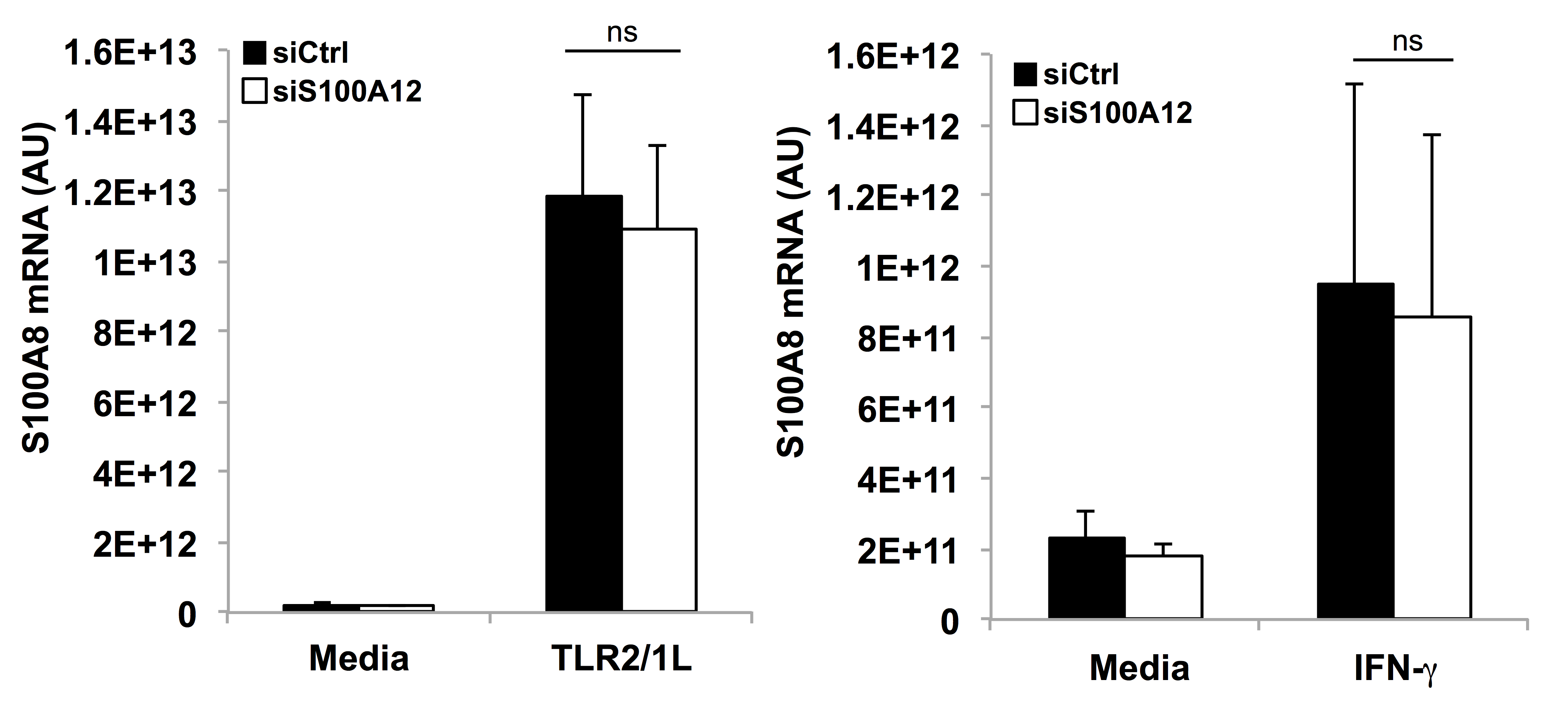

Supplement: S6 Fig — MDMs were transfected with siRNA specific for S100A12 (siS100A12) or non-specific (siCtrl) and subsequently treated with TLR2/1L (n = 9) or IFN-γ (n = 5) for 24 hours. S100A8 mRNA was assessed by qPCR (mean FC ± SEM). (TIFF) [file ppat.1005705.s008.tiff]
